# Transient Facial Nerve Paralysis (Bell's Palsy) following Intranasal Delivery of a Genetically Detoxified Mutant of *Escherichia coli* Heat Labile Toxin

**DOI:** 10.1371/journal.pone.0006999

**Published:** 2009-09-16

**Authors:** David J. M. Lewis, Zhiming Huo, Susan Barnett, Ingrid Kromann, Rafaela Giemza, Eva Galiza, Maria Woodrow, Birgit Thierry-Carstensen, Peter Andersen, Deborah Novicki, Giuseppe Del Giudice, Rino Rappuoli

**Affiliations:** 1 St George's Vaccine Institute, St George's University of London, London, United Kingdom; 2 Novartis Vaccines, Siena, Italy; 3 Staten Serum Institute, Copenhagen, Denmark; Charité-Universitätsmedizin Berlin, Germany

## Abstract

**Background:**

An association was previously established between facial nerve paralysis (Bell's palsy) and intranasal administration of an inactivated influenza virosome vaccine containing an enzymatically active *Escherichia coli* Heat Labile Toxin (LT) adjuvant. The individual component(s) responsible for paralysis were not identified, and the vaccine was withdrawn.

**Methodology/Principal Findings:**

Subjects participating in two contemporaneous non-randomized Phase 1 clinical trials of nasal subunit vaccines against Human Immunodeficiency Virus and tuberculosis, both of which employed an enzymatically inactive non-toxic mutant LT adjuvant (LTK63), underwent active follow-up for adverse events using diary-cards and clinical examination. Two healthy subjects experienced transient peripheral facial nerve palsies 44 and 60 days after passive nasal instillation of LTK63, possibly a result of retrograde axonal transport after neuronal ganglioside binding or an inflammatory immune response, but without exaggerated immune responses to LTK63.

**Conclusions/Significance:**

While the unique anatomical predisposition of the facial nerve to compression suggests nasal delivery of neuronal-binding LT–derived adjuvants is inadvisable, their continued investigation as topical or mucosal adjuvants and antigens appears warranted on the basis of longstanding safety via oral, percutaneous, and other mucosal routes.

## Introduction

Mucosal immunization via intranasal route has received much attention in recent years [1] due to potential advantages of needle-free delivery and enhanced mucosal immune responses against infections such as influenza or Human Immunodeficiency Virus (HIV), which can be enhanced by co-administration of effective mucosal adjuvants such as mutants of *Escherichia coli* heat labile toxin (LT) [2]. However, this immunization approach raised concerns after a strong epidemiological association was reported [3] between facial nerve paralysis (Bell's Palsy) and intranasal administration of inactivated influenza virosome vaccine *“NasalFlu”* containing an enzymatically active mutant LT adjuvant [4]. Retrospective analysis did not identify relative contributions of vaccine components [3], and *NasalFlu* was withdrawn. We report two cases of transient Bell's Palsy following nasal administration of a genetically detoxified LT adjuvant (LTK63) [2] during two Phase 1 vaccine trials.

## Methods

### Objectives and participants

The cases reported here occurred during two contemporaneous, open-label, Phase 1 clinical vaccine trials of safety and immunogenicity at the same clinical site (St George's Vaccine Institute, London). No formal sample size calculations were made, as is usual in this early stage of evaluation. Full details of trials outcomes are in preparation for publication. Briefly, nine BCG vaccine-naive healthy subjects aged 18–45 were entered into a non-randomized Phase 1 trial of a nasal *Mycobacterium tuberculosis* subunit vaccine [5] (full protocol available on ClinicalTrials.gov NCT00440544: http://clinicaltrials.gov/ct2/show/NCT00440544, EudraCT: 2005-005983-10). The first four subjects received two nasal immunizations (days 0 and 56) with 100 µg *Mycobacterium tuberculosis* Ag85B-ESAT6 fusion protein alone, and the subsequent five subjects received two nasal immunizations (days 0 and 56) of 100 µg *Mycobacterium tuberculosis* Ag85B-ESAT6 fusion protein mixed with 30 µg LTK63. After the event reported here the trial was terminated prior to recruitment of subsequent planned groups.

In the contemporaneous trial, 30 healthy subjects aged 18–45 were entered into a non-randomized Phase 1 clinical trial of a nasal HIV glycoprotein-140 subunit vaccine [6] (full protocol available on ClinicalTrials.gov NCT00369031: http://clinicaltrials.gov/ct2/show/NCT00369031, EudraCT: 2005-005140-81), and allocated to one of three consecutively-recruited groups (10 per group) to receive three intranasal immunisations (days 0, 28, and 56) with either: 100 µg HIV glycoprotein-140 mixed with 30 µg LTK63, 100 µg HIV glycoprotein-140 alone, or 30 µg LTK63 alone. All subjects received two intramuscular immunizations with 100 µg HIV glycoprotein-140 mixed with MF59 adjuvant [7] on days 112 and 196. Following the case reported here 4 subjects in the group receiving 30 µg LTK63 alone nasally underwent only one nasal immunization, but did receive two intramuscular immunizations. The subject reported here had no further immunizations after the first nasal immunization with 30 µg LTK63 alone.

### Collection, processing, and analysis of serum and nasal lavage samples for immune responses to LTK63

Blood and nasal samples were obtained at pre-defined time points before and after immunization. Blood was collected by antecubital fossa venepuncture, serum separated and frozen at −20°C. Nasal lavage with 5 mL sterile water was performed via urinary catheter placed the anterior nares bilaterally, and samples frozen at −80°C. IgG and IgA antibodies to LTK63 were detected by indirect ELISA [8], expressed in µg/mL using a human immunoglobulin standard (NIBSC, UK).

### Investigations for alternative causes of facial nerve paralysis

Pre- and post-immunization serum or plasma samples were retrospectively analysed for evidence of the most common conditions associated with Bell's palsy including serology/PCR/antigen detection of infection with Human Immunodeficiency Virus, Epstein-Barr Virus (EBV), Varicella zoster virus (VZV), *Borrelia burgdorferi*, measles, mumps, Herpes simplex 1 and 2 (HSV) and cytomegalovirus (CMV); or autoimmune diseases including antinuclear antibodies (ANA), anti-neutrophil cytoplasmic antibodies (ANCA) and Angiotensin Converting Enzyme (ACE) levels. Both subjects declined radiological examinations.

### Ethics

All subjects provided written informed consent before undergoing screening or participation in the trials, which were both approved by St George's Research Ethics Committee and UK Medicines and Healthcare products Regulatory Agency. In addition the two subjects reported here provided additional written consent for publication of details which may include identifying information.

## Results

### Case 1

A healthy 29 year old, white male enrolled into nasal *Mycobacterium tuberculosis* subunit vaccine trial 2005-005983-10. He reported an uncomplicated nasal procedure age 6, insertion of tympanic membrane grommets in childhood, mild conjunctivitis when pollen count high, mild itching after prolonged grass contact, but no seasonal rhinitis, neurological disease, migraine or regular medications. Screening HIV, hepatitis B and C serology, hematology and biochemistry were negative or normal. Mantoux test and ‘QuantiFERON-TB Gold’ (Cellestis, UK) were negative for latent tuberculosis. Medical examination including ear, nose and throat (ENT) and cranial nerves was normal apart from scar on right tympanic membrane. Chest radiograph was normal. He received 100 µg *Mycobacterium tuberculosis* Ag85B-ESAT6 fusion protein and 30 µg LTK63, in 300 µL physiological buffer, by passive instillation of drops divided equally between right and left anterior nares. A diary was issued to record symptoms.

On day 1 he recorded mild malaise, ENT discomfort, and cough. Examination on day 7 was normal. Between days 34–43 he recorded mild itching of eyes and took loratidine. Examination on day 43 was normal. Between days 45–48 he experienced mild coryza and nasal discharge treated with acetaminophen. Examination on day 56 was normal, and he received second nasal immunization with Ag85B-ESAT6 and LTK63 as before. Between days 57–61 took loratidine for a sensation of fluid in right ear. On day 58 he experienced a migraine-like visual aura bilaterally, lasting 20 minutes. On day 60 he noticed mild right sided post-auricular pain, followed by abnormality of taste, worsening over day 61. On day 62 (5 days after second immunization) he developed right-sided facial asymmetry and increased tear production.

On examination on day 64 he was afebrile, cardiovascular, respiratory, abdominal examination normal, no mastoid tenderness, rashes or peri-auricular vesicles. ENT normal except clear fluid behind non-inflamed right tympanic membrane. Sense of smell normal on testing. Cranial nerves 2–6 normal, corneal reflex intact, pupils equal and responsive, fundoscopy normal. House-Brackmann [9] grade V signs of right lower motor neurone facial nerve palsy present, with inability to taste salt, sour, and bitter on right anterior portion of tongue, and profuse tear production (seen in 67% of Bell's palsies [10] as orbicularis oculis dysfunction prevents tear transport to lacrimal sac, without hypersecretion). Marked Bell's phenomenon (upward gaze upon attempting to close affected eye) observed, confirming peripheral lesion [11]. Hearing and balance normal, Rinne's normal, Weber localized to right (seen in Bell's palsy, also compatible with observed middle ear fluid). Cranial nerves 9–12 normal. Sensation over head and neck normal. He was prescribed prednisolone, omeprazole, aciclovir (8 days), eye drops & patches according to UK guidance [12]. Palsy was graded III on day 70, and II by day 78 without increased tearing, and steroids discontinued. Symptoms of right retro-auricular pain and abnormality of taste recurred between days 87–89 (30–32 after second immunization) without facial paralysis. He recommenced prednisolone and aciclovir for 11 and 7 days respectively. On day 91 examination was normal except slight abnormality of taste. Examination on days 126 and 225 (168 days after second immunization) were normal.

There were no clinically significant abnormalities of hematology and biochemistry throughout study. Screening and day 225 samples were tested for: HIV 1&2 antibody, p24 antigen (negative); VZV (IgM negative, IgG positive, no rise in titer); *B. burgdorferi* (IgG and IgM negative); measles, mumps (IgG positive, no rise in titer); HSV 1 and 2 (IgG negative); CMV (IgG and IgM negative); ANA, ANCA (negative). ACE 22 U/L day 56, and 16 day 91 (normal range 8–52). Anti-BV Nuclear Antigen positive on screening and on day 225, no rise in titer; anti-EBV Early Antigen IgG 52 at screening rising to 150 on day 225; anti-EBV Early Antigen IgM 23·5 (weak positive) at screening and 14·2 (negative) on day 225. This pattern suggests possibility of EBV infection in months before screening.

### Case 2

A healthy 20 year old, white female enrolled into nasal HIV glycoprotein-140 subunit vaccine trial 2005-005140-81. She gave no history of neurological or otolaryngology disease, was taking no regular medications except oral contraceptives. Screening HIV, hepatitis B and C serology, plasma HIV nucleic acid, hematology and biochemistry panels, blood and urine pregnancy tests were negative or normal. Physical examination was normal including ENT and cranial nerves. She received 30 µg of LTK63 (as a nasal placebo) in 284 µL physiological buffer, by passive instillation of drops divided equally between right and left anterior nares. A symptoms diary was issued.

On days 2 and 3 she recorded mild malaise, nasal congestion and headache, resolved with ibuprofen. Examination on day 3 was normal. Between days 8–11 she reported mild headache, resolved by stat acetaminophen and pseudoephedrine. On day 28 she was afebrile, physical examination normal; hematology and biochemistry normal. Her scheduled second (and subsequently third) immunization was cancelled due to Bell's palsy affecting Case 1. On days 37–40 she reported mild nasal congestion and discharge, on day 43 mild cough, and on days 44–45 left sided shoulder/neck pain “like a pulled muscle”, and took diclofenac. On day 44 she developed left retro-auricular pain, stinging of left eye with increased tearing, and jaw paresthesia, followed by facial asymmetry and difficulty closing left eye. A local Emergency Room diagnosed Bell's palsy, but gave no treatment. On day 45 abnormal taste sensation developed and she attended the trial site. There was no history of trauma, headache, fevers, aura/visual disturbance, or abnormality of smell. She was afebrile; cardiovascular, respiratory, abdominal, ENT examinations normal; no mastoid tenderness, rashes or peri-auricular vesicles. Normal sense of smell on testing; cranial nerves 2–6 intact; pupils equal and responsive, fundoscopy normal. A grade IV left lower motor neurone facial nerve palsy was present with difficulty distinguishing salt, sour and bitter taste on anterior portion of tongue, and increased tearing. Hearing and balance, Rinne's and Weber tests normal. Gag reflex present but decreased on left. Cranial nerves 11–12 normal, with appearance of tongue deviating to right (feature of Bell's palsy due to mouth asymmetry, not twelfth nerve defect). Sensation over head and neck normal. She was prescribed prednisolone, omeprazole, aciclovir, eye drops & patches [12]. Grade II weakness was present on day 44, with reduced taste sensation. A Consultant Neurologist confirmed the diagnosis on day 48. Palsy almost completely resolved by day 50, and steroids were discontinued, with complete recovery by day 58. She was discharged on day 214 with no further relapse.

There were no clinically significant abnormalities of hematology and biochemistry throughout study. Screening and day 224 samples were tested for: HIV 1&2 antibody, p24 antigen and HIV nucleic acid PCR (negative); VZV (IgG positive, no rise in titer, IgM negative); *B. burgdorferi* (IgG and IgM negative); measles (IgG positive, no rise in titer); mumps, HSV 1 and 2 (IgG negative); EBV (EBNA IgG positive, no rise in titer, EBV Early Antigen IgG and IgM negative); CMV (IgG and IgM negative); ANA, ANCA (negative). ACE 42 U/L day 27, 24 day 55 (normal range 8–52).

### Immune responses to LTK63

**Figure 1:**
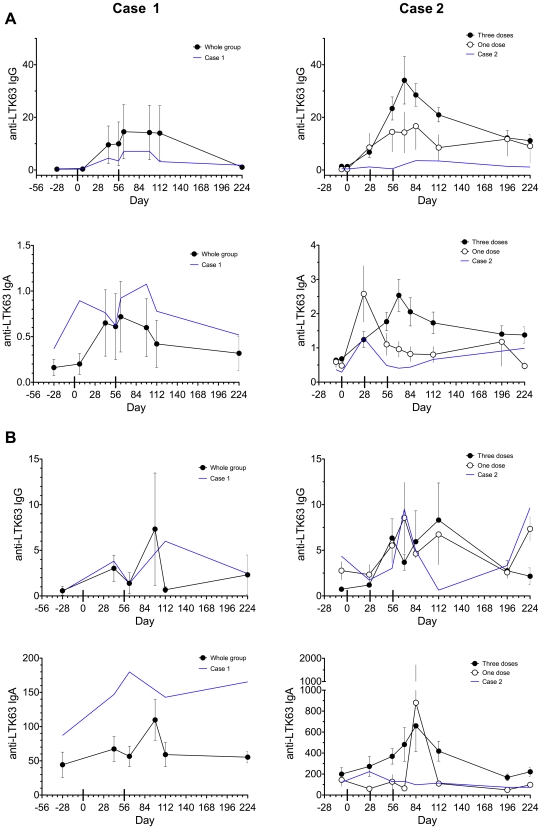
Serum and nasal lavage antibody response to LTK63. Anti-LTK63 specific IgG and IgA in serum (µg/mL, (A)) and nasal lavage (ng/mL, (B)) measured by ELISA. Time course for individual response of Case 1 (left figures) and Case 2 (right figures) shown as blue lines. Response of remainder of group shown as group mean: for Case 1 (closed circles) n = 4; for Case 2 (who received only one dose) n = 15 receiving three doses (closed circles), and n = 4 receiving one dose (open circles). Error bars indicate SEM. Immunization days 0, 28, and/or 56 indicated by broad ticks crossing x-axis.

LT is a potent mucosal antigen [13] therefore antibody responses to LTK63 were measured using an in-house ELISA on serum and nasal lavage samples obtained from subjects before and after immunization. IgG and IgA responses to LTK63 were observed after each immunization (Figure 1), and although numbers in each group are small, responses in cases experiencing Bell's palsy were generally comparable with group mean.

## Discussion

We report two cases of Bell's palsy temporally associated with nasal administration of non-toxic LTK63, possibly a result of transient interference with peripheral nerve function due to accumulation of LTK63 molecules, or inflammation arising from immune response to LTK63, following ganglioside binding and retrograde neuronal transport; or another unknown mechanism. Both subjects rapidly recovered without long-term sequelae. The situation of our subjects prospectively recording the progressive symptoms and signs of Bell's palsy from a clearly defined time-point offered an unique opportunity to study in some detail the pathophysiology of the neurological disturbance, in contrast with most clinical studies in which symptoms are recalled retrospectively, and in which there is no clearly defined starting point.

Bell's palsy arises from facial nerve compression within the facial canal, or acute demyelination, usually associated with herpes infection, but also other conditions including parenteral immunization, infections, tumors and autoimmune diseases [11]. Cholera toxin (CT) undergoes retrograde axonal transport by binding neuronal gangliosides [14], a property shared by LT, but unlike tetanus toxin CT cannot cross synapses [14]. Considerable attention has focused on safety issues associated with possible olfactory nerve transport of CT/LT molecules to the CNS [15], a single cell-system with no synapse outside the CNS. Whereas human olfactory nerve endings are poorly accessible to passive instillation of drops into the anterior nares, facial nerve secretomotor fibers richly supply nasal mucosa, and can transport molecules to the geniculate ganglion via the nerve of the pterygoid canal and greater petrosal nerve. The sequential appearance of classical symptoms and signs of Bell's palsy, without olfactory disturbance we observed, are consistent with progressive interference of peripheral (not central nervous system) facial nerve function by a process initiated during retrograde axonal transport. Intriguingly, secretomotor symptoms preceded both cases, which is of interest as antecedent symptoms of “upper respiratory tract infection” are reported in 20% of Bell's palsies [16], and often ascribed as a cause rather than a consequence of the neurological defect. The involvement of trigeminal and glossopharyngeal nerves in Case 2 is well recognised [16] possibly due to spread of the causal agent (usually a herpes virus) via superficial greater petrosal nerve [17], which connects facial, trigeminal and glossopharyngeal nerves. The migraine-like aura without headache in Case 1 is interesting as carotid plexus autonomic innervation joins the greater petrosal nerve, and release of vasoactive neuropeptides from trigeminal and parasympathetic perivascular fibers has been associated with migraine [18]. The onset of symptoms on day 44 and 60 (with a possible relapse 31 days after second immunization in Case 1) is consistent with the highest risk of palsy between 31 to 60 days after *NasalFlu* [3], suggesting a common pathogenesis.

An annual Bell's palsy incidence of 32 per 100,000 was reported in a European population [10], We observed two cases of Bell's palsy in 25 subjects, receiving 59 LTK63 30 µg doses, making a chance association highly unlikely. Only 13 cases per 10,000 were estimated within 91 days after vaccination with *NasalFlu* [3], and no toxicity was seen during 42 day follow-up in a Phase I trial of a Chiron (now Novartis) intranasal inactivated influenza vaccine [19] in which 18 subjects received two doses of 30 µg LTK63 alone; a further 16 two doses of 30 µg LTK63 with influenza antigens and particulate biovector; and another 30 receiving two doses of 3 or 10 µg LTK63 with antigens and biovector. It is possible that our free suspension of LTK63 allowed more molecules to bind neuronal gangliosides than when complexed with virosomes (*NasalFlu*) or biovectors (Chiron-Novartis vaccine), and the absence of symptoms in 18 subjects receiving LTK63 alone in the Chiron nasal vaccine trial is not significant (Fisher's exact test: *p* = 0.52; Odds Ratio 3.2 (0.15–68).

It was speculated [20] that reactivation of herpes infection may have been induced by toxicity of the LT used in *NasalFlu* (which induced diarrhea when given orally [4]) as extensive preclinical evaluation, including primates, was unremarkable [21]. We followed standard UK guidance and both subjects received antiviral and steroid therapy, and rapidly made a full recovery, although it is not possible to determine whether the treatment affected the course of the disease. Both cases were seronegative for HSV (although Case 1 had some weak serological evidence of asymptomatic EBV infection in the months prior to recruitment), and LTK63 is rendered completely non-toxic by an amino acid substitution in the enzyme active site that abolishes ADP-ribosylation [2]. Our vaccines were produced under GMP conditions, free of contaminants including lipopolysaccharide, and nasal testing in rabbits, including detailed CNS histology, revealed no localized or generalized toxicity. Extensive preclinical experience with nasal LTK63 [2] has demonstrated mucosal adjuvanticity without toxicity, including macaques [6], Guinea Pigs [22], and mice [5].

In conclusion, the unique anatomical predisposition of the facial nerve to compression within the facial canal suggests that nasal administration of neuronal-binding LT-derived molecules is inadvisable. However, the extensive clinical safety record of oral [13] and percutaneous [23] administration of CT/LT molecules provides a rationale for their continued investigation as potent mucosal and topical vaccine antigens and adjuvants. The lack of reported facial nerve paralysis following nasal immunization in subjects not receiving LT adjuvants supports the continued exploration of nasal delivery as an effective and needle-free route of immunization against mucosally-acquired infections as influenza and HIV.
